# Postoperative Outcomes of Unroofing Technique for Anomalous Aortic Origin of a Coronary Artery With Intramural Course: A Single-Center Study

**DOI:** 10.7759/cureus.110800

**Published:** 2026-06-13

**Authors:** Daichi Hosokawa, Mika Saito, Kanako Kishiki, Tomomi Uyeda, Satoshi Yazaki, Tadahiro Yoshikawa, Yuya Komori, Masatoshi Shimada, Naoki Wada, Hikoro Matsui

**Affiliations:** 1 Department of Pediatric Cardiology, Sakakibara Heart Institute, Tokyo, JPN; 2 Department of Pediatric Cardiovascular Surgery, Sakakibara Heart Institute, Tokyo, JPN; 3 Department of Pediatric Cardiovascular Surgery, St. Marianna University School of Medicine, Tokyo, JPN

**Keywords:** anomalous aortic origin of a coronary artery, aortic regurgitation, commissure-intramural distance, intramural course, unroofing technique

## Abstract

Background

Anomalous aortic origin of a coronary artery (AAOCA) with an intramural course is a recognized cause of sudden cardiac death. Unroofing is the standard surgical treatment; however, postoperative aortic regurgitation (AR) and its anatomical predictors remain incompletely understood.

Methods

We retrospectively analyzed 16 patients who underwent unroofing for AAOCA with an intramural course. The commissure-intramural distance was measured on preoperative computed tomography (CT), and its association with postoperative AR progression was evaluated.

Results

During a median follow-up of six months, there were no deaths or coronary-related reinterventions. Postoperative AR progression was observed in 38% of patients, and all cases were trivial to mild. The commissure-intramural distance was significantly shorter in patients with AR progression than in those without (2.66 mm vs. 4.81 mm, p = 0.047).

Conclusions

Unroofing for AAOCA with an intramural course was associated with favorable early clinical outcomes in our cohort. A shorter commissure-intramural distance on preoperative CT may be associated with postoperative AR progression. Given the small sample size, these findings should be interpreted as exploratory and hypothesis-generating.

## Introduction

Anomalous aortic origin of a coronary artery (AAOCA) is a significant cause of sudden cardiac death in young individuals, highlighting the importance of early diagnosis and appropriate intervention. Despite its low prevalence (0.1-1% of the population), certain anatomical features - particularly an intramural course, as well as an interarterial course, slit-like orifice, and steep take-off angle - are considered “malignant” due to their association with ischemia and lethal arrhythmias [1]. Surgical intervention, particularly the unroofing technique for AAOCA with an intramural course, is frequently performed to reduce the risk of adverse cardiac events. Although previous studies have demonstrated favorable outcomes, postoperative complications - especially aortic regurgitation (AR), the most common valvular complication - remain an important concern [2-4]. Therefore, this study aimed to evaluate surgical outcomes with a focus on postoperative AR and to investigate the association between preoperative CT-based anatomical parameters, specifically the commissure-intramural distance, and postoperative AR using 3D geometric analysis.

In particular, because postoperative AR progression is presumed to be related to whether the unroofing procedure affects the aortic commissure, we measured the commissure-intramural distance as a quantitative indicator of this anatomical relationship.

This article was previously presented as a meeting abstract at the 9th World Congress of Pediatric Cardiology and Cardiac Surgery on December 10, 2025.

## Materials and methods

We retrospectively analyzed 16 patients who underwent the unroofing for AAOCA with an intramural course at our institution between March 2008 and August 2024. Clinical records and imaging data were retrospectively reviewed to collect patient demographics, including age, sex, body weight, mode of diagnosis, and preoperative symptoms.

Preoperative investigation included stress myocardial scintigraphy and exercise electrocardiography. The presence and severity of preoperative AR were evaluated by transthoracic echocardiogram. All patients underwent enhanced CT. Operative details included the level of cutback (above the commissure or above the raphe), whether the incised edges were tacked to the aortic wall, whether the commissure was directly manipulated, and whether concomitant procedures were performed. Postoperative outcomes included symptom changes, ischemic findings, AR severity, coronary-related and non-coronary related reinterventions, and follow-up duration. The primary outcome was postoperative AR progression, defined as new-onset AR or worsening of pre-existing AR on follow-up echocardiography.

Morphological assessment for the intramural course of AAOCA was performed on preoperative enhanced CT using a workstation (Aquarius iNtuition (version 4.4.13. P3A; TeraRecon, Inc., Foster City, CA). We measured the commissure-intramural distance, defined as the shortest distance from the aortic commissure to the intramural segment of the coronary artery.

Measurements were obtained on multiplanar reformatted images of the aortic root. The superior edge of the commissure was identified on axial and sagittal views focused on the commissure, and the shortest distance to the intramural coronary segment was measured on the coronal view. The commissure-intramural distance was measured by consensus between two observers according to a predefined imaging protocol. Formal assessment of interobserver variability was not performed.

**Figure 1:**
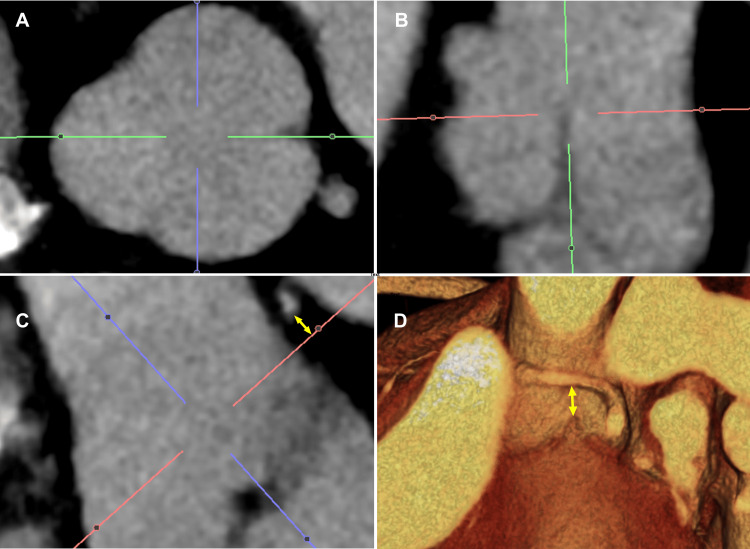
Preoperative CT measurement of the commissure-intramural distance. Multiplanar reformatted (MPR) enhanced CT images demonstrating measurement of the commissure-intramural distance. (A) Axial view. (B) Sagittal view. (C) Coronal view used for measurement of the shortest distance between the superior edge of the aortic commissure and the intramural segment of the anomalous coronary artery (double-headed arrow). (D) Three-dimensional volume-rendered reconstruction illustrating the anatomical relationship between these structures. The double-headed arrow in panel D represents the same commissure-intramural distance measured on the coronal MPR image shown in panel C.

The association between postoperative AR progression and the commissure-intramural distance was evaluated using the Mann-Whitney U test. Statistical analyses were performed using JMP version 11.2.0 (SAS Institute Inc., Cary, NC) (Figure 1).

Continuous variables are presented as median values with interquartile ranges (IQRs), and a p-value of <0.05 was considered statistically significant.

This study was approved by the Institutional Review Board of Sakakibara Heart Institute (approval no. 25-071), and written informed consent was obtained from all participants.

## Results

Among the 16 patients (male:female = 14:2), the median age at surgery was 13.1 years (range, three to 49 years). Diagnoses included anomalous origin of the right coronary artery (AAORCA) in 10 (63%), anomalous origin of the left coronary artery (AAOLCA) in five (31%), and left coronary artery origin from the non-coronary sinus in one (6%). Six patients (38%) were asymptomatic, while others presented with exertional dyspnea, chest pain, or nausea, ventricular fibrillation (VF) or cardiopulmonary arrest (CPA), syncope, and resting chest pain. Stress myocardial scintigraphy was positive in six of 10 patients and exercise electrocardiography in two of six. Preoperative AR was absent in 15 patients and trivial in one (Table 1).

**Table 1:** Baseline characteristics of the study cohort. Patient demographics, anatomical diagnosis, preoperative symptoms, and preoperative testing results are summarized. AAOLCA, anomalous aortic origin of left coronary artery; AAORCA, anomalous aortic origin of right coronary artery; AR, aortic regurgitation; CPA, cardiopulmonary arrest; VF, ventricular fibrillation

Characteristic	Value
Age at surgery, years	13.1 (3-49)
Male sex	14 (87.5%)
Body weight, kg	52.1 (17.6-66.5)
AAOCA type	
AAORCA	10 (63%)
AAOLCA	5 (31%)
LCA from non-coronary sinus	1 (6%)
Preoperative symptoms - asymptomatic	6 (38%)
Dyspnea/chest pain/nausea	4 (25%)
VF/CPA	3 (19%)
Syncope	2 (12%)
Resting chest pain	1 (6%)
Stress myocardial scintigraphy	Positive 6/10
Exercise ECG	Positive 2/6
Preoperative AR	
None	15 (94%)
Trivial	1 (6%)

Cutback above the commissure or raphe was performed in 15 patients, whereas in one patient with a short intramural segment, the incision was terminated before reaching the commissure. The incised aortic wall was excised as extensively as possible, and the edges were tacked to the aortic wall using 7-0 or 6-0 polypropylene sutures in 11 patients. Direct manipulation of the commissure occurred in only one case. Concomitant procedures included right pulmonary artery graft interposition, partial atrioventricular septal defect repair, and pulmonary valve replacement, each in one patient.

**Figure 2:**
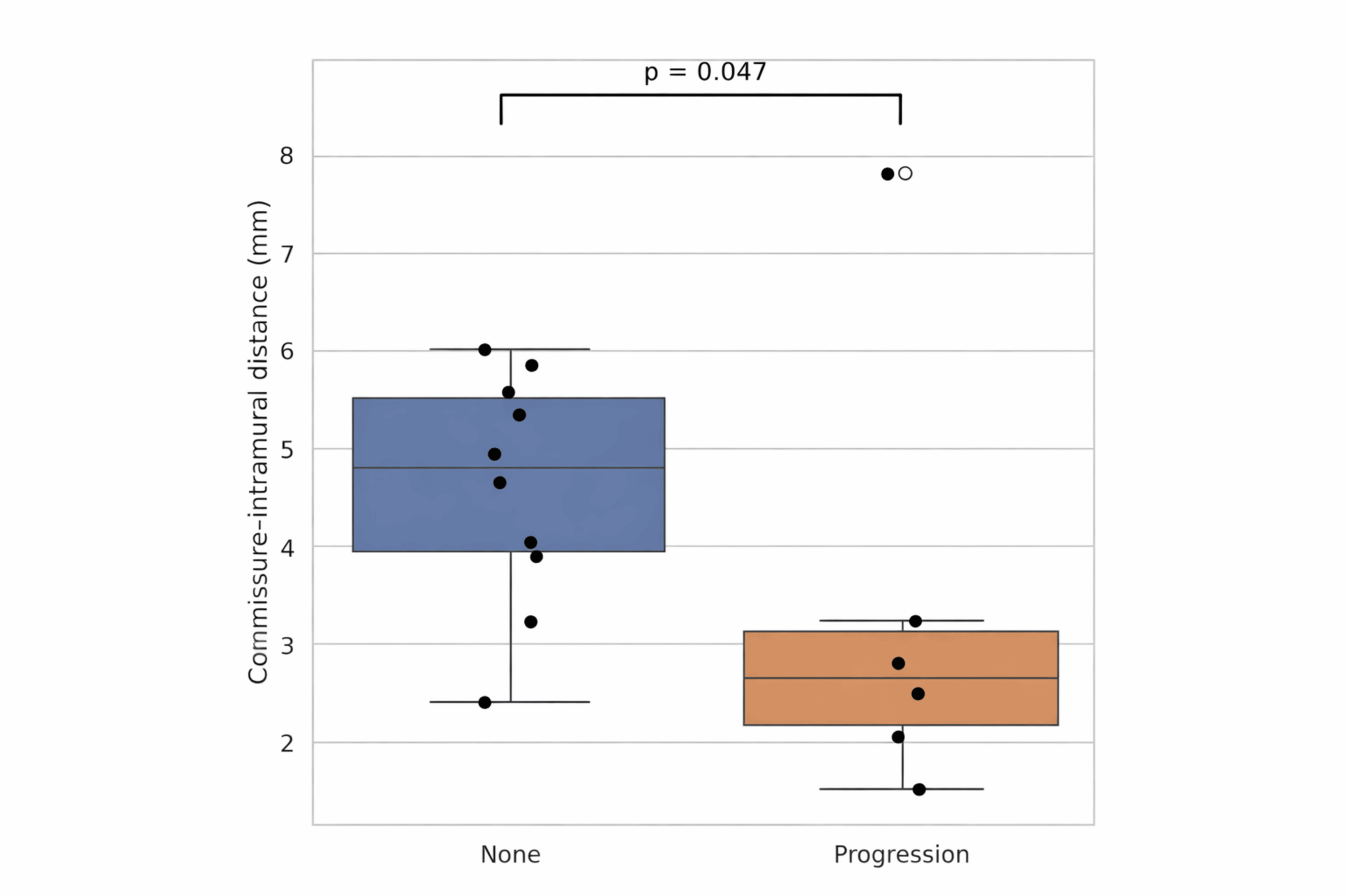
Comparison of the commissure-intramural distance between patients with and without postoperative aortic regurgitation progression. Boxplots illustrate the commissure-intramural distance measured on preoperative CT in patients with (orange) and without (blue) postoperative aortic regurgitation (AR) progression. Individual data points are overlaid to show the distribution within each group. Median values and interquartile ranges are displayed.

The median follow-up was six months (range, one month to 16 years). There were no deaths or coronary-related reinterventions; one patient required ICD implantation for a non-coronary indication. Symptoms resolved in 15 patients, while one had exertional chest pain despite no abnormalities on coronary angiography or stress scintigraphy, with gradual improvement over time. At last follow-up, AR was absent in 10 patients, trivial in three, and mild in three (38%, 6/16), with no interventions required. AR progression occurred in six patients, including five patients with no preoperative AR. Most cases demonstrated central AR, whereas eccentric jets were observed in a patient with a bicuspid aortic valve and in one undergoing concomitant partial atrioventricular septal defect repair, the latter of which showed transient mild AR that subsequently improved. The only patient who underwent commissural manipulation progressed from no AR to mild AR. The commissure-intramural distance measured on CT (4.36 (2.66-5.72) mm) was significantly longer in the non-AR progression group than in the AR progression group (4.81 vs. 2.66 mm, p = 0.047), suggesting an association with postoperative AR progression (Table 2, Figure 2). 

**Table 2:** Comparison of postoperative aortic regurgitation and the commissure-intramural distance. Postoperative aortic regurgitation (AR) severity and the commissure-intramural distance measured on preoperative CT are compared between patients with and without AR progression. Continuous variables are presented as median values with interquartile ranges (IQRs), and categorical variables are shown as counts with percentages. Statistical analysis was performed using the Mann-Whitney U test.

Postoperative AR	N (total = 16)	The commissure-intramural distance	p-value
None	10 (62%)	4.81 mm (3.91-5.58)	0.047
Progression	6 (38%)	2.66 mm (2.06-3.24)	-
Trivial	3 (19%)	-	-
Mild	3 (19%)	-	-

## Discussion

Overall outcomes in the context of guideline-supported management

In this study, unroofing for AAOCA with an intramural course was associated with excellent clinical outcomes, with no mortality or coronary-related reinterventions. These findings are consistent with current guideline-supported surgical management and prior reports demonstrating the safety and efficacy of unroofing [5,6]. Even patients presenting with VF/CPA achieved symptom resolution and improvement in ischemic findings, supporting the effectiveness of surgical intervention in high-risk patients. Favorable outcomes were observed in both AAOLCA and AAORCA, in line with expanding surgical indications for AAORCA in the contemporary guidelines [1,7].

Postoperative AR: incidence and surgical considerations

Postoperative AR remains a relevant concern following unroofing. In our cohort, AR occurred in 38% of patients; however, all cases were trivial to mild, clinically insignificant, and required no intervention, consistent with previous reports [3,4,8]. Although commissural manipulation has been suggested as a potential contributor to AR, its impact remains controversial. In our study, the only patient who underwent commissural manipulation developed mild AR, whereas other technical variations were not clearly associated with AR progression. These findings suggest that, while surgical technique may play a role, it alone may not fully explain the development of postoperative AR.

Association between preoperative CT measurements and postoperative AR

A key finding of this study is that the commissure-intramural distance was significantly shorter in patients with postoperative AR progression. A short distance may indicate that the intramural segment terminates near the commissure, making the commissural support more susceptible to alteration during unroofing and potentially affecting aortic valve leaflet mobility. Previous studies have suggested that intramural segment length and commissural relationships may influence postoperative AR; however, no consistent quantitative thresholds have been established [9]. Our CT measurements provide additional quantitative evidence supporting this association and suggest that this parameter may help identify patients at higher risk of postoperative AR.

Clinical implications, limitations, and future directions

Our findings support the use of unroofing not only for AAOLCA, as recommended by current guidelines, but also for symptomatic AAORCA, AAORCA with positive ischemia testing, and AAORCA with anatomical risk factors such as an intramural course [1,7]. Although postoperative AR occurred in a subset of patients, all cases were mild and clinically insignificant, reinforcing the safety of unroofing in our cohort. Importantly, the commissure-intramural distance may be associated with postoperative AR. A shorter distance may warrant greater attention to commissural preservation during surgery and may help refine preoperative risk stratification and individualized surgical planning. As more Japanese AAOCA surgical data accumulate, incorporating objective preoperative CT-based anatomical parameters into decision-making may further optimize outcomes.

Despite these important findings, several limitations should be acknowledged. This study is limited by its single-center, retrospective design and small sample size, which restricts statistical power.

Variations in surgical technique and surgeon experience may have influenced outcomes. In addition, the clinical significance of mild AR remains uncertain due to limited follow-up in some patients. In addition, the commissure-intramural distance was measured by consensus between two observers; however, formal assessment of interobserver variability was not performed.

Therefore, the observed association between commissure-intramural distance and postoperative AR progression should be interpreted with caution. These findings are exploratory and should be considered hypothesis-generating. 

Future multicenter studies with larger cohorts are needed to further evaluate the association between the commissure-intramural distance and postoperative AR progression, establish clinically relevant thresholds, and clarify the role of commissural preservation techniques. Integrating preoperative CT findings with intraoperative anatomy and postoperative functional assessment may further improve surgical decision-making.

## Conclusions

Unroofing for AAOCA with an intramural course showed favorable early clinical outcomes in our cohort, with no mortality or coronary-related reinterventions. Although postoperative AR progression occurred in a subset of patients, all cases were trivial to mild and clinically insignificant. A shorter commissure-intramural distance on preoperative CT may be associated with postoperative AR progression and warrants further investigation in larger studies.
